# Matching on poset‐based average rank for multiple treatments to compare many unbalanced groups

**DOI:** 10.1002/sim.9192

**Published:** 2021-09-16

**Authors:** Margherita Silan, Giovanna Boccuzzo, Bruno Arpino

**Affiliations:** ^1^ Department of Statistical Sciences University of Padua Padua Italy; ^2^ Department of Statistics, Computer Science, Applications University of Florence Florence Italy

**Keywords:** matching, multiple treatments, neighborhood effect, poset

## Abstract

In this article, we propose an original matching procedure for multiple treatment frameworks based on partially ordered set theory (poset). In our proposal, called matching on poset‐based average rank for multiple treatments (MARMoT), poset theory is used to summarize individuals' confounders and the relative average rank is used to balance confounders and match individuals in different treatment groups. This approach proves to be particularly useful for balancing confounders when the number of treatments considered is high. We apply our approach to the estimation of neighborhood effect on the fractures among older people in Turin (a city in northern Italy).

## 1 INTRODUCTION

The ideal situation for drawing causal inference about a given treatment is represented by randomized trials, however, quite often in many fields, randomization of subjects into different treatment groups is infeasible.^1^ The use of observational data represents a challenge because of selection bias, that happens when the treated group of subjects differs systematically from the control group, according to confounders that also affect the outcome or are associated to it. Indeed, the distribution of confounders among the treatment groups may differ considerably, creating what is called an unbalanced situation. Thus, in these cases the crucial question is whether differences with respect to the outcome between treatment groups can be attributed to the treatment itself, rather than to differences between subjects' characteristics in the groups.^2^ This is why methodological techniques usually applied to solve this issue are focused on reaching the balance of confounders' distribution across the treatment groups.

Widely used methods to balance the distributions of observed characteristics among treatment groups are those based on the propensity score, that is, the probability to be treated conditional on a set of independent variables. Propensity score matching, for example, consists in matching individuals in different treatment groups based on their propensity score.^3^ This method enables to adjust for confounders, and compare only comparable groups of subjects, with similar distribution of the observed characteristics. Several studies deal with the use of propensity score techniques in the presence of a dichotomous treatment, where only two groups of subjects need to be balanced with respect to confounders. The use of propensity score techniques in multiple treatment frameworks is less straightforward. In the literature there are a few works that estimate the effect of nondichotomous treatments,^4^, ^5^, ^6^, ^7^, ^8^, ^9^ comparing three or four treatment groups. However, using propensity score matching is complicated with multiple treatments, especially when the number of treatment groups is higher than three, because of methodological, interpretative and computational issues. For instance, it is more difficult to identify a common support for all the treatment groups that allow to properly compare them. Also the computation of the propensity score is less trivial, especially the model specification. Moreover, the complexity of the matching algorithm increases with the number of treatment group considered.

The method we propose, that we label matching on poset‐based average rank for multiple treatments (MARMoT), consists in a multiple matching based on partially ordered sets (poset). We test the MARMoT approach with simulations, and prove its utility in balancing the observed confounders among groups. Our simulation study considers 23 treatment groups and shows more than satisfactory results, indeed, the MARMoT approach highly improves the balance of confounders, even starting from strongly unbalanced situations. As far as the empirical application is concerned, we observed MARMoT performance with real data considering 10, 23, and 70 treatment groups. Even with real data the results are satisfactory, especially with 10 and 23 treatment groups, while in the last case there is still room for improvement. As a matter of fact, it is quite common in public health applications to deal with high number of treatments, for instance in the assessment of hospitals' performance.^10^

In our empirical analyzes, we estimate the neighborhood effect on hospitalized fractures among older people residents in Turin, a city in the north of Italy. The focus of this paper is on comparison of neighborhoods with different compositions, given that individuals with different fracture risk factors may live in different areas. Our matching technique enable us to adjust for confounders using a poset‐based average rank in a multiple treatments framework, even when the number of treatment groups (neighborhoods) is very high. The main contributions of this paper thus consist in the proposal of this new matching approach based on poset theory, its validation in a simulation study and its application to estimate the neighborhood effect on hospitalized fractures among individuals aged sixty or more, based on three different geographical partitions.

In Section 2, we describe the case study, the data analyzed, the three different geographical partitions considered, and the confounders we measure. The methods to balance the observed confounders in multiple treatments frameworks are described in Section 3, followed by a brief introduction to poset theory, and an in‐depth explanation of our proposal. In Section 4, we describe the design and the results of a simulation study that we performed to test the reliability of our original proposal. Section 5 illustrates the empirical application with real data, comparing different geographical partitions and a sensitivity analysis that shows strengths and limitations of the MARMoT approach.

## 2 CASE STUDY

During the last 20 years, there has been growing interest in the effects of context on individuals' lives,^11^ prompting important new research in social epidemiology. Such effects are usually called “neighborhood effects” and were defined by Oakes^1^ as the independent causal effects of neighborhoods on a given health or social outcome. In the literature, the term neighborhood is often used to delineate individuals' immediate residential environments and the material and social characteristics of these environments that presumably have an impact on personal outcomes.^12^ Risk factors for health attributable to neighborhood include deprivation, walkability, air pollution, crime, and social cohesion.^11^

Our interest in the neighborhood effect on hospitalized fractures among individuals aged 60 and more stems from a real need expressed by Turin's Epidemiological Service. Neighborhoods may affect fracture rates among older people in two main ways: they may be difficult to walk around, or have inadequate street lighting, and thus increase the risk of falls. In addition, people living in the area may be discouraged from engaging in physical activity, and their muscle tone and bone structure may consequently deteriorate.^13^, ^14^ The focus here is on people aged 60 and more, partly because of their greater exposure to hospitalized fracture, and also because they tend to be a more stable resident population. Indeed, some researchers have found older people more susceptible to experience neighborhood effects because they spend more time in their neighborhoods than younger people.^15^ Older people are also less likely to move house (the annual rate for the observed population was only around 1%).

### 2.1 Turin longitudinal study

The data used in our analyzes come from a longitudinal study conducted in Turin, that gave rise to an integrated database, which combines administrative data flows on residents drawn from censuses and population registry with health data flows (hospital discharge records, prescription charges and exemptions, and territorial drug prescriptions). The hospital discharge records contain information on the patient's diagnosis, admission mode (emergency, compulsory, voluntary), and date of admission and discharge. The prescription charges database lists all exemptions from payment of health services to which some patients are entitled due to chronic conditions or low income. The territorial drug prescriptions database contains details of prescribed drugs, the quantities involved, and their classification (based on their therapeutic, pharmacological and chemical properties). The census data includes not only basic demographic data, such as age, sex, and place of birth, but also some important information about individuals' socioeconomic status, such as their occupation, education, home ownership, and family composition.

All these different data sources have been pooled together over time. Starting with the censuses and population registers available in 1971, Turin's residents have been registered and tracked as a historical migration dataset, considering all movements of individuals living in Turin for at least one day from 1971 onwards.^16^ Several other data sources were added over time, such as the cause of death archives in 1971, the cancer registry in 1985, the hospital discharge records in 1995, drug prescriptions data in 1997, and so forth.

### 2.2 Examined population

The study population consists of all individuals included in the 2001 population census, aged 60 or more at December 31, 2001. In order to be able to collect information on possible confounders represented by past health‐related information, we focus on individuals who have been living in Turin between January 1, 1997 and December 31, 2001. We measure the outcome, that is, hospitalized fractures, during the year following the census (ie, 2002). We therefore limit our analyzes to individuals who have been living in Turing for the whole year 2002. In the empirical analyzes, we focus on assessing the differences in the proportion of individuals experiencing at least one hospitalized fracture in 2002 among individuals living in different neighborhoods at the time of the 2001 census.

### 2.3 Neighborhoods

The city of Turin can be split into 10 districts, 23 areas, or 94 zones. The three partitions are characterized by different living conditions (deprivation, walkability, crime, and social cohesion) and population features, but the three geographical layers are only partially hierarchical. For instance, the same zone may belong to two or more areas, or districts.

The ten districts have an average population of 22 583 residents, with the least populated accounting for 10 608 individuals, and the most populated for 33 072 individuals. The populations of the 23 areas range between 3584 and 18 089 residents, with an average of 9819. The number of individuals living in each zone varies even more.

In our empirical analyzes, we compare proportions of hospitalized fractures among neighborhoods considering the three geographical partitions. In the case of the 94 zones, however, we excluded the neighborhoods with small sizes. More specifically, we excluded zones with a population of less than 625 individuals (corresponding to the first quartile of the distribution of the population size of zones). The number of individuals living in the zones thus discarded accounts only for the 3% of the whole population, and the final number of zones considered is 70. For the sake of brevity, in the simulations we focus on the intermediate partition, that is the city divided into 23 areas.

### 2.4 Variables

Based on the literature on neighborhood effects on older people's health,^12^ we consider the following variables as possible confounders: gender, age (considering five‐year‐age brackets: 60‐64, 65‐69, 70‐74, 75‐79, 80, and over), region of birth, family composition, educational attainment, last known occupational condition, and home ownership. The region of birth distinguishes between individuals born: in Piedmont (the region to which Turin belongs); in other regions of Northern Italy; central Italy; Southern Italy or islands; or outside Italy. The variable representing family composition combines marital status with the number of components: living alone; married and living only with a partner (family of two); unmarried and not living alone (family of two or more); married and living in a family of more than two people. The last known occupational situation is a variable obtained from the census data from 1971 to 2001, and aims to capture the last type of occupation prior to retirement. This was not possible for some individuals because they were already retired in 1971 (and in all other censuses where they appear), or they were not working for other reasons. The occupation variable distinguishes between the just‐mentioned case and home‐makers, entrepreneurs, white‐collar workers, and manual workers.

The percentage of hospitalized fractures in 2002 is equal to 0.9% of Turin residents aged 60 or more, with some differences between neighborhoods. The percentage of hospitalized fractures, in fact, varies between 0.67% and 1.18% across the different areas. Although the outcome may be considered as a rare event, this fact does not represent an obstacle for our analysis. In fact, the matching method we propose does not require modeling the outcome and focuses on attaining an acceptable balancing of the distribution of confounding variables among the different neighborhoods.^17^

## 3 METHODS

Let $𝒯=\{t_{1},t_{2},\ldots ,t_{K}\}$ be the treatment support for *K* multiple treatments. Under the standard *stable unit treatment value assumption (SUTVA)* and given *K* treatments, we have a set of K potential outcomes, $𝒴=\{Y_{i}(t_{1}),Y_{i}(t_{2}),\ldots ,Y_{i}(t_{K})\}$ and only one of them is observed for each individual *i*. Let $s_{1}$ and $s_{2}$ represent two subgroups of treatments (two groups of neighborhoods) such that $s_{1},s_{2}\subset 𝒯$, and $s_{1}\cap s_{2}=Ø$. Next, let $\left|s_{1}\right|$ and $\left|s_{2}\right|$ be the cardinality of $s_{1}$ and $s_{2}$, respectively.^18^ Following Lopez and Gutman,^5^ we can formulate a general causal estimand that compares average potential outcomes between two groups of treatments:

(1) $$ATE_{s_{1},s_{2}}=E\left[\frac{\sum_{t\in s_{1}}Y_{i}(t)}{\left|s_{1}\right|}-\frac{\sum_{t^{\prime }\in s_{2}}Y_{i}(t^{\prime })}{\left|s_{2}\right|}\right].$$

In Equation (1), the expectation is over all units, i=1,…,N, and the summation is over the potential outcomes of a specific unit. Several estimands can be considered as special cases of (1), including pair‐wise ATEs where $s_{1}$ and $s_{2}$ are singleton, that is, they include a specific treatment only. Pair‐wise ATEs would contrast two pairs of neighborhoods and one could consider all possible pairs. In the context of our application, one may consider a special case of (1), where central and peripheral neighborhoods are contrasted. In fact, the researcher or a policy maker might be first interested in answering the question of whether central neighborhoods are less risky in terms of older people's hospitalized fractures as compared to peripheral neighborhoods. In a second step, then one could compare specific neighborhoods by using pair‐wise ATEs.

In neighborhood research it is usually of interest to address the question of whether living in any other neighborhood than a specific one would improve health. Therefore, in this study, we focus on an estimand that compares every treatment group (neighborhood) with the rest of treatments (the rest of the city), and we define it as the average treatment effect of a specific neighborhood against the rest of the city (ATENC). Let *t* represents a generic element of 𝒯, and 𝒞 represents the other treatments, 𝒞=𝒯−{t}, ATENC is:

(2) $${\mathrm{ATENC}}_{t,𝒞}=E\left[Y_{i}(t)-\frac{\sum_{c\in 𝒞}Y_{i}(c)}{\left|𝒞\right|}\right].$$

Our ATENC is transitive, thus it is possible to directly compare ATENCs for different neighborhoods.

Identification of causal estimands such as those derived from (1), usually rely on the assumptions of positivity and unconfoundedness. The positivity or sufficient overlap assumption, that is 0<p(t|Y(1),…,Y(K),X)<1, ∀t∈{1,…,K}, implies that there are no values of pretreatment confounders that could occur only among units receiving one of the treatments. The treatment assignment is unconfounded, that is p(t|Y(1),…,Y(K),X)=p(t|X), ∀t∈{1,…,K}, which implies that the set of observed pretreatment confounders, X, is sufficiently rich such that it includes all variables directly influencing both treatment assignment and the outcome; in other words, there is no unmeasured confounding.

Matching and weighting using the generalized propensity score (GPS), that is, the conditional probability of being in a particular treatment group given pretreatment variables,^19^ are common approaches to compare multiple treatments. Applications of the GPS matching or weighting remain largely scattered in the literature, with few applications involving three (or four) treatments.^4^, ^5^, ^6^, ^8^, ^9^, ^20^, ^21^ Other methods that have been tested and compared include regression adjustment, marginal mean weighting through stratification, and “doubly robust” estimators.^7^, ^22^

None of these methods are practical, however, if the number of treatments is higher than three (in general, the higher is number of treatments, the more complex and less practical the mentioned methods are). Some important assumptions (such as the overlap) become difficult to satisfy, and estimating propensity scores becomes computationally demanding and may turn in the estimation of very small probabilities of belonging to the several treatment groups.

An alternative approach, that does not require estimating treatments probabilities is template matching. This method can handle the balance of several treatments.^10^ With this approach, a sample of individuals represented in all the treatment groups, the template, is selected to guarantee comparability of the individuals in all treatment groups included in the analysis. Then the matching algorithm matches individuals from all treatment groups with the template, and all other individuals are discarded. The analysis is thus restricted to individuals belonging to the common support of confounders across all the treatment groups. This enables to manage many treatments, but limits the generaliability of the analyzes, and results may heavily depend on the choice of the template.

In the following, we describe our original alternative approach to deal with confounders balance when comparing many treatments which involves matching on a score (average rank) obtained using partially ordered sets (poset) theory. This approach, that we label MARMoT (matching on poset‐based average rank for multiple treatments) allows us to make the distribution of confounders similar across many treatment groups. We will rely on the most commonly employed measure of comparability of the treatment groups (balance) in terms of each confounder X, the absolute standardized bias (ASB). Because our variables are all represented by a series of dummy variables (one for each level of the variables), other, more refined, measures of balance would not relevant. In addition, ASB has been found superior to other measures in its ability to predict the bias of causal effects estimators.^23^ ASB will be measured as:

(3) $$ASB=\frac{\left|{\bar{X}}_{t}-\bar{X}\right|}{\sqrt{\frac{S_{t}^{2}}{2}+\frac{S^{2}}{2}}},$$

where $\bar{X}$ and ${\bar{X}}_{t}$ are the means of the variable *X* of individuals living respectively in the whole city, and in the neighborhood *t*; and *S* and $S_{t}$ are the standard deviations of the variable *X* for individuals living respectively in the whole city and in the neighborhood *t*.

### 3.1 Matching on poset‐based average rank for multiple treatments (MARMoT)

Here, we first briefly introduce key concepts in poset theory (for a more detailed presentation, see Part A of the Supplementary Material), and then we describe our matching approach.

#### 3.1.1 Poset theory and average rank computation

Poset theory is a theoretical field between graph theory and discrete mathematics that quickly developed after the 1970s thanks to technological advances that made greater computational efforts manageable.^24^ A partially ordered set (poset) is a set of elements where a binary relation that indicates an order can be traced. The word “partially” refers to the fact that not every pair of elements is necessarily comparable.

People in a population can be ranked and ordered using a single variable. For instance, the level of education enables two different individuals to be arranged in an order. The set of observed characteristics of each individual is called “profile”. If the comparison is drawn using several variables, it may be that some elements are neither equal nor ordered, in which case they are defined as incomparable.^25^ The word “partially” is added to “ordered set” when some elements of the population are incomparable, so the order relation has to be changed to a partial order relation, which takes the incomparability of the elements into account.

Comparing the individuals in a population gives rise to a list of comparabilities and incomparabilities, which can be represented graphically using a Hasse diagram. This diagram represents the elements in a poset: each node is an element, two or more equal elements still form one node, and every line segment is an order relation between comparable objects. Dichotomous, categorical and discrete variables may be considered in a poset. However, in order to contain the complexity of the poset, it is recommended to reduce each variable to a few meaningful classes.

Linear extensions are all the possible rankings of elements in the poset that respect its comparabilities (the connections in the Hasse diagram) and incomparabilities.^24^, ^25^ The average rank (AR) of a node represents the mean of all the ranks that the element occupies in all possible linear extensions, starting from the known order relations. Thus, the AR is a single value for each element in the set that describes the relative position of a given element with respect to the rest of the population. This score can be normalized in the interval [0, 1].

AR's involvement in the MARMoT approach serves the same goal of the GPS, that is, the purpose is to reduce data dimensionality and balance observable individuals' characteristics. Similar to the GPS, there is no need to attach a substantial interpretation to AR values for the purpose of matching on it.

If the number of individuals and variables increases, the linear extensions become too many to be examined thoroughly, and it becomes computationally almost impossible to calculate the exact value of the AR. However, satisfactory approximations of the number of linear extensions of a poset have been suggested.^26^, ^27^ Researchers have used two main approaches to obtain a computationally efficient calculations of the AR, by sampling linear extensions,^28^, ^29^ or by using approximation formulas, such as the local partial order model,^30^ or the one based on mutual probabilities.^27^ We rely on the De Loof's approach^31^ because it provides better results than other methods in terms of accuracy with a large sample size.^31^ More specifically, we calculate approximated ARs using the R package deloof developed by Caperna^32^, ^33^ that can deal with large datasets.^34^, ^35^

### 3.2 The matching

We use our MARMoT technique to summarize confounders, applying a poset‐based AR calculated for each individual. As such, the individuals' characteristics are summarized by unique AR values, and individuals who have similar ARs have profiles that are nearby in the poset linear extensions. AR enables us to proceed with a matching whereby each individual in a given neighborhood is associated to individuals with similar ARs in all the other neighborhoods, and those who cannot be matched are discarded in order to respect the overlap condition and make all neighborhoods simultaneously comparable.

Once the AR has been computed (resulting in *R* different values, where R≤N), the first step is to build a frequency table, as Table 1, where each row corresponds to a specific observed value of the AR ($AR_{r}$, r=1,…,R), and each column represents a treatment group ($t_{k}$, k=1,…,K). In other words, $f_{r,k}$ is the number of individuals with AR equal to $AR_{r}$ and assigned to the treatment group $t_{k}$. For instance, supposing that $AR_{1}=0$, $f_{1,1}$ is the number of individuals with AR=0 living in neighborhood 1.

**TABLE 1:** An example of the frequency table involved in the matching of the MARMoT approach

AR	$t_{1}$	$t_{2}$	…	$t_{k}$	…	$t_{K}$
$AR_{1}$	$f_{1,1}$	$f_{1,2}$	…	$f_{1,k}$	…	$f_{1,K}$
$AR_{2}$	$f_{2,1}$	$f_{2,2}$	…	$f_{2,k}$	…	$f_{2,K}$
⋮	⋮	⋮	⋱	⋮	⋱	⋮
$AR_{r}$	$f_{r,1}$	$f_{r,2}$	…	$f_{r,k}$	…	$f_{r,K}$
⋮	⋮	⋮	⋱	⋮	⋱	⋮
$AR_{R}$	$f_{R,1}$	$f_{R,2}$	…	$f_{R,k}$	…	$f_{R,K}$

In order for each value of the AR to be represented equally in all the treatment groups, the desired result would be a table where $f_{r,1}=f_{r,2}=\cdots =f_{r,k}=\cdots =f_{r,K}=f_{r}$, ∀k=1,…,K in every row *r*. Thus, for every row, we should choose the most appropriate frequency $f_{r}$ for each AR value to be imposed in the balanced population. In the artificial final population, the distribution of AR values will be balanced in all the treatments groups so as to balance all confounders too. At the end of the matching procedure, each $AR_{r}$ value will be present in the balanced population $K*f_{r}$ times, with $f_{r}$ individuals in each of the K treatment groups. The value for $f_{r}$ may be chosen according to different criteria. For example, it may be the maximum, the mean, the median or the minimum of the frequencies in row *r*. We define the reference $f_{r}$ as the median of the frequencies in row *r*:

(4) $$f_{r}=\left\{\begin{array}{cc} 1\; & \text{if}\;\text{median}(f_{r,1},f_{r,2},\ldots ,f_{r,K})=0\\ \text{median}(f_{r,1},f_{r,2},\ldots ,f_{r,K})\; & \text{otherwise}. \end{array}\right.$$

Instead of discarding all the AR values with $\text{median}(f_{r,1},f_{r,2},\ldots ,f_{r,K})=0$, we set the minimum value of $f_{r}$ at 1 in order to have a matched population that includes all the profiles in the real population. The choice of the value for $f_{r}$ affects both the final dimension of the balanced dataset, and the performance of the MARMoT method in terms of balance, as will be described in more detail in Section 5.1.5. Having established the frequency that each value of AR should have in each treatment, the algorithm proceeds in three different ways, depending on the dimensions of $f_{r,t}$ and $f_{r}$, for every *r* and every *t*:

1. if $f_{r,t}=f_{r}$: all individuals with $AR_{r}$ (that is the AR value in row r) in the treatment group $t_{k}$ are copied in the final dataset;
2. if $f_{r,t}\neq f_{r}$ and $f_{r,t}\neq 0$: a random sample of size $f_{r}$ with replacement is selected from the group of individuals with the same $AR_{r}$ in the treatment group *t*, and included in the final dataset;
3. if $f_{r,t}=0$: a random sample with replacement of size $f_{r}$ is selected drawing from the individuals with an AR close enough (with a given tolerance) to $AR_{r}$ in treatment group *t*, and included in the final dataset. If there are no individuals within the chosen tolerance levels of AR, then all individuals with an AR equal to $AR_{r}$ have to be discarded.

While points (1) and (2) only imply matching individuals with identical AR values, point (3) is the trickiest part because it involves inexact matching, and possibly excluding some individuals from the final dataset. We define the tolerance interval as $\left[AR_{r}-\frac{S_{AR}}{4};AR_{r}+\frac{S_{AR}}{4}\right]$, considering as a caliper the value $\frac{S_{AR}}{4}$, where $S_{AR}$ is the AR's sample standard deviation, similar to recommendations on caliper setting in the propensity score matching literature.^36^, ^37^ Thus, if all frequencies $f_{.,t}$ in treatment group *t* that correspond to AR values included in the interval $\left[AR_{r}-\frac{S_{AR}}{4};AR_{r}+\frac{S_{AR}}{4}\right]$ equal 0, subjects with AR value equal to $AR_{r}$ will be discarded in all the treatments groups. This criterion ensures that the overlap assumption is respected.

As a final remark, the MARMoT method is strongly influenced by five fundamental aspects:

1. the number of individual's characteristics considered, which directly affects the number of AR values (the number of rows in Table 1);
2. the number of the levels of categorical variables and the inclusion of discrete variables that may increase the complexity of the poset (and the number of rows in Table 1);
3. the number of treatments, that is, the number of columns in Table 1;
4. the size of the total population, *N*, which corresponds to $N=\sum_{r}\sum_{t}f_{r,t}$; and
5. the choice of $f_{r}$ that, as discussed above, affects both the final dimension of the balanced dataset and the quality of matches.

Given the population size, an increase in one of the first three factors is expected to cause an increase of inexact matching cases with a consequent worsening of the balancing. Instead, a higher population size is expected to improve the algorithm's performance. All these aspects will be examined in a sensitivity analysis in Section 5.

Once the MARMoT algorithm has matched the individuals and balanced the confounders, the ATENC can be estimated.

## 4 SIMULATION STUDY

Before using the MARMoT method to estimate the neighborhood effect on real data, we tested it with some simulations in two different scenarios considering a situation with 23 treatments. The R code used for the simulation study is reported in Part B of the Supplementary Material.

### 4.1 Simulation design

To keep our simulation close to the real application of interest, we considered the real population of Turin and the individuals' observed characteristics. Starting from the seven confounders described in Section 2, we simulated the treatment allocation according to two different scenarios. Since the computation of the AR depends only on the confounders (which come from the observed population and are not simulated artificially), and not on the treatment, AR values computed directly on the observed data could be used, meaning that they were based exclusively on the real population, not on simulated values.

In the first scenario, the treatment allocation equation is simple and close to the real situation. The treatment is generated through a multinomial logistic model, taking neighborhood 20 (the one with the lowest crude hospitalized fractures rate) as the reference category.

In order to choose values for the coefficients, we estimated a multinomial logistic model on the real data using the whole population. These coefficients were perturbed by adding a random value from a uniform distribution between −0.01 and +0.01, and rounded up or down to three decimals. The small range for the uniform distribution guarantees coefficients' perturbation without compromising the balance of confounders that remains close to the real situation.

The second scenario envisages a more complex treatment allocation equation, which includes all the interactions between the seven variables considered. As in the first scenario, the choice of parameters for these treatment allocation equations was based on those estimated by a multinomial logistic model, perturbed by a uniform distribution between −0.1 and +0.1 (also in this case the range of the uniform distribution is chosen as a trade‐off between perturbation and balance), and rounded up or down to three decimals.

### 4.2 Results

The main results of the simulations are shown in Table 2, where column S indicates the above‐described treatment allocation scenarios (coded as 1 for the linear and additive, and 2 for the one with interactions). The first part of Table 2 shows the results of the simulation as described in the previous section, the differences between the scenarios and the differences in the distribution of the individuals among the neighborhoods.

**TABLE 2:** Results of simulations in the two treatment (T) scenarios

	S	1	2	3	4	5	6	7	8	9	10	11	12	13	14	15	16	17	18	19	20	21	22	23
		Simulation design																						
(a)	1	3.49	3.98	4.28	2.86	4.31	4.65	3.54	3.93	3.59	5.91	5.70	7.76	7.83	5.47	5.83	5.71	1.92	4.63	4.25	2.24	1.59	2.13	4.39
(a)	2	4.11	3.58	4.03	3.54	4.18	4.84	4.02	4.44	4.08	4.54	6.04	7.44	6.15	5.61	6.35	6.30	1.83	3.82	4.63	2.18	1.93	1.86	4.50
		**Balance before and after matching**																						
(b)	1	15.4	12.6	15.8	10.2	15.9	11.2	11.2	5.1	9.6	6.3	9.7	11.3	5.7	3.5	14.8	10.1	10.2	7.6	16.8	10.3	10.3	11.5	16.5
(c)	1	4.3	1.5	4.0	0.2	0.8	0.6	0.8	0.0	1.1	0.1	0.1	0.3	0.0	0.0	3.2	1.4	3.7	2.3	4.5	4.5	6.7	8.6	3.8
(d)	1	9.8	8.0	10.1	1.6	5.8	3.7	5.8	0.5	3.2	0.0	5.7	2.8	0.9	1.0	9.3	6.6	6.0	6.2	10.4	8.6	7.9	7.9	10.0
(e)	1	1.2	0.0	2.3	0.0	0.0	0.0	0.0	0.0	0.0	0.0	0.0	0.0	0.0	0.0	0.6	0.0	1.6	0.0	0.7	0.8	2.3	5.0	0.5
(b)	2	15.3	13.8	14.8	14.0	16.9	13.8	12.5	7.6	16.8	10.8	9.8	14.9	8.1	7.1	15.9	10.0	13.5	12.6	14.9	10.0	16.4	11.7	16.2
(c)	2	5.1	1.9	4.6	0.1	1.1	1.0	0.5	0.0	1.4	0.0	0.8	0.0	0.0	0.0	2.2	1.2	5.1	3.9	5.1	5.1	5.8	9.2	5.5
(d)	2	12.0	9.0	10.0	7.0	9.1	7.6	6.1	1.4	8.4	2.1	5.1	8.4	1.0	0.3	11.8	6.1	8.1	8.6	11.0	8.1	11.8	8.8	12.8
(e)	2	1.5	0.0	2.4	0.0	0.0	0.0	0.0	0.0	0.0	0.0	0.0	0.0	0.0	0.0	0.7	0.0	2.5	0.5	1.0	1.1	2.0	5.7	0.3

*Note*: (a) mean percentage of the distribution of individuals among neighborhoods in 1000 iterations; (b) mean number of ASB higher than 5% before matching, for each neighborhood; (c) mean number of ASB higher than 5% after matching, for each neighborhood; (d) mean number of ASB higher than 10% before matching, for each neighborhood; and (e) mean number of ASB higher than 10% after matching, for each neighborhood.

We examined the initial balance in the two scenarios in all 1000 replications using the ASB. Having 23 neighborhoods and seven variables (for a total of 24 levels), we chose to summarize the information by computing the minimum, first quartile, mean, median, third quartile, and maximum of all the ASB, counting ASB values over 5% and 10% for each replication. The means of these values among all 1000 simulations before and after the balancing procedure for each scenario are given in Table 3. The balance was much improved in both scenarios after the matching procedure, which fixed even extremely unbalanced situations. After matching, the mean number of ASB over 10% corresponded to one tenth of the number beforehand. The central part of Table 2 shows the means (over the 1000 replications) of the number of ASB higher than 5% and 10% before and after adjusting for each neighborhood. From these results, we can see that our MARMoT method greatly improves the balance of confounders among neighborhoods: it achieves a five‐fold reduction in the number of ASB over 5%, and an almost 10‐fold reduction in those over 10%, in both scenarios.

**TABLE 3:** Mean of ASB summary statistics in the first and second scenarios before and after balancing among 1000 simulations

Scenario	Balance	Min	First quartile	Median	Third quartile	Max	Mean	Over 5%	Over 10%
First	Before	0.01	1.98	4.43	9.59	63.72	8.01	252	132
	After	0	0.58	1.30	2.59	16.95	2.12	52	15
Second	Before	0.02	2.42	5.63	11.89	68.41	9.10	297	175
	After	0	0.62	1.37	2.71	16.84	2.24	60	18

## 5 EMPIRICAL RESULTS AND SENSITIVITY ANALYSIS

In this section, we use our MARMoT technique to estimate neighborhood effects considering 10 districts, 23 smaller areas and 70 zones. Moreover, in order to explore limits and strengths of our proposal, we conduced also a sensitivity analysis to observe MARMoT‐s performance depending on meaningful modifications of the fundamental aspects enlisted in Section 3.2.

### 5.1 Sensitivity analysis

As explained in Section 3.2, there are five aspects that affect MARMoT performance: the number of considered confounders, the number of levels of categorical variables, the number of treatments, the size of the total population and the choice of the frequency reference $f_{r}$ for every row. In order to assess their impact on MARMoT's performance as a balancing procedure, we modified each of this aspect at the time and we assessed MARMoT performance, using as a basis for comparison the application (also reported in Table 4, as bold text) on the whole population with 23 treatments that considers all the variables and all the levels we described as confounders in Section 2.4. The findings are summarized in Table 4 in terms of balance and some diagnostic measures about the MARMoT procedure. In order to summarize the information coming from all the computed ASB, we computed the minimum, first quartile, mean, median, third quartile, and maximum of all the ASB, counting ASB values over 5% and 10%. Regardless of the modification made in the sensitivity analysis, the balance is always computed on the basis of the same variables and levels (seven variables for a total amount of 24 levels). Moreover, in order to assess the MARMoT procedure more broadly, we reported also some additional diagnostic measures such as: the percentage of inclusion (% of inclusion) that represents the proportion of individuals of the initial population included in the final balanced sample; the average number of times each individual is repeated in the final population (Rep. mean); the maximum number of times that an individual is duplicated in the balanced dataset (Rep. max), the size of the neighborhood (which is the same in all neighborhoods after the balance, while it is reported the average in the rows that represent the situation before the balance, marked with ${}^{*}$) and the size of the total population. We suggest the applied researcher to always report these diagnostics to improve the transparency of the analyzes.

**TABLE 4:** Sensitivity and empirical analysis' results regarding the balance, in terms of ASBs' summary statistics, and some diagnostic measures about the MARMoT procedure

			Balance (ASB)								Diagnostic measures				
		Before or after matching	Min	First quart.	Median	Third quart.	Max	Mean	Over 5%	Over 10%	% of inclusion	Rep. mean	Rep. max	Neigh. size	Pop. size
Lower number of the number of individual characteristics	Age removed	After	0.001	0.229	0.554	1.331	10.100	1.099	20	1	56.805	1.609	29	8,974	206,402
	Occupation removed	After	0.003	0.187	0.472	1.261	19.575	1.450	42	12	57.028	1.592	35	8,912	204,976
	Region of birth removed	After	0.001	0.221	0.494	1.307	42.179	1.753	45	17	57.349	1.602	39	9,021	207,483
Change the levels of categorical variables	Aggregated levels	After	0.000	0.226	0.636	2.567	32.480	2.885	84	51	57.513	1.589	31	8,973	206,379
	Splitted levels	After	0.012	0.908	1.946	4.024	26.634	3.463	103	44	67.708	2.117	39	14,073	323,679
Different number of treatments	10	Before	0.012	1.689	3.563	7.587	56.207	7.242	101	46				22,582.8*	225,828
		After	0.000	0.192	0.427	0.937	8.948	0.846	5	0	61.920	1.662	32	23,239	232,390
	**23** *(basis for comparison)*	**Before**	**0.072**	**1.934**	**4.198**	**9.774**	**63.763**	**7.938**	**248**	**132**				9,818.6*	**225,828**
		**After**	**0.003**	**0.482**	**1.169**	**2.330**	**15.802**	**1.973**	**51**	**11**	**62.342**	**1.792**	**79**	**10,969**	**252,287**
	70	Before	0.008	2.556	5.723	12.287	105.132	10.020	914	522				3,132.8*	219,294
		After	0.008	1.539	3.523	7.075	55.625	5.725	624	265	67.436	2.824	56	5,966	417,620
Different size of the total population	75%	Before	0.015	2.045	4.545	9.348	66.669	8.116	256	128				7,364*	169,371
		After	0.008	1.789	3.585	7.187	48.074	5.766	216	96	60.170	1.944	79	8,605	197,915
	50%	Before	0.008	2.094	4.689	9.973	65.561	8.192	261	138				4,909.3∗	112,914
		After	0.000	1.296	2.710	4.995	30.273	4.166	138	57	61.234	2.166	79	6,512	149,776
	25%	Before	0.003	2.002	4.556	9.810	64.812	8.064	261	135				2,454.7*	56,457
		After	0.000	0.855	1.917	3.788	20.973	3.092	92	35	66.527	2.758	120	4,504	103,592
	50% = treatment size	Before	0.013	1.895	4.231	9.591	61.731	8.004	253	130				4,909	112,907
		After	0.028	0.977	2.024	3.983	25.487	3.397	103	42	57.748	2.354	31	6,673	153,479
Different specification for $f_{r}$	$f_{r}=\max(f_{r,1},f_{r,2},\ldots ,f_{r,K})$	After	0.000	0.495	1.120	2.262	16.361	1.953	53	10	91.381	3.368	170	30,220	695,060
	$f_{r}=\min(f_{r,1},f_{r,2},\ldots ,f_{r,K})$	After	0.000	0.000	0.000	0.000	0.000	0.000	0.000	0.000	8.728	1.098	6	941	21,643

*Note*: Neighborhood sizes marked with ${}^{*}$ are averages.

#### 5.1.1 The number of individual's characteristics

Starting from the list of seven variables described in Section 2.4, we run the MARMoT procedure three times excluding a different variable at the time: age, last known occupational condition and region of birth. These three variables have five levels each, in this way the algorithm deals with the same number of theoretical profiles (1200 instead of 6000 as in the basis for comparison). Results are summarized in the first three rows of Table 4. In general, with a reduced number of variables to be balanced among neighborhoods, ASBs after matching are lower than in the basis for comparison for all the variables, with the exception of course of the variable that was excluded from the balancing procedure. The balance of the removed variable is improved by the MARMoT procedure anyway, but it still shows high levels of ASB in some cases. Differences observable in Table 4 are due to differences in the initial balance of the excluded variable before the MARMoT procedure: indeed, for instance, age was already almost well balanced in the initial situation, thus, after MARMoT, balance is very satisfactory.

#### 5.1.2 The number of levels of categorical variables

In order to explore the behavior of the MARMoT procedure with different numbers of levels of categorical variables, we considered two scenarios: one in which several levels were aggregated (864 theoretical profiles) and one in which some levels were disaggregated (12 800 theoretical profiles) with respect to the basis of comparison (6000 theoretical profiles). Both scenarios in Table 4 show worse ASBs compared to the basis for comparison, however their ASB values tell two completely different stories. In the first scenario, the one with aggregated levels, all the high ASB values are placed in correspondence of the levels that are aggregated, while in all the others the balance is satisfactorily achieved. On the other hand, in the second scenario the high ASB levels are scattered among all the variables.

The conclusions that we draw from these scenarios give us some important guidelines regarding the number of variables and levels that should be selected to be considered in the MARMoT procedure. Indeed, with 23 treatments and 225 828 subjects in the whole population, when the number of theoretical profiles is lower than in the basis for comparison, variables and levels considered in the balancing procedure are perfectly balanced, while with a higher number of theoretical profiles the algorithm provides a not perfectly balanced dataset. In other words, there is a delicate equilibrium between the number of theoretical profiles considered in the balancing procedure and the quality of the balance in the final dataset that is reflected in a trade‐off between the need to include all the possible confounders and the quality of the resulting balance.

#### 5.1.3 The number of treatments

As explained in Section 2.3, Turin may be divided into three geographical partitions: 10 districts, 23 areas, and 70 zones. As a basis of all the comparisons in this section we have chosen the 23 areas partition, but we examined MARMoT's performance also with a lower (10) and a higher (70) number of treatments. The procedure to balance the 10 districts took less than 18 minutes, the one for the 23 areas took 36 minutes, and the balancing of the 70 zones took 116 minutes. Table 4 shows that the MARMoT method substantially reduces the ASB with all three partitions considered, but slightly less successfully in the case of the 70 zones.

The balance in the 10 treatments case is easier to reach than for the other partitions, indeed the mean ASB after the balancing procedure is lower than 1%, while in the 23 treatments case the mean ASB is almost 2%.

The mean ASB computed for the 70 zones decreases from around 10% before the MARMoT adjustment to 5.7% in the matched dataset. Before matching, the majority of the 70 considered zones had at least half of the computed ASB higher than 5%, while after MARMoT adjustment the number of these zones is halved and the number of zones with half of the computed ASB higher than 10% is null. The percentage of zones with a quarter of the computed ASB higher than 10% decreases from around 63% before the matching, to 21.4% after the MARMoT adjustment.

**FIGURE 1:**
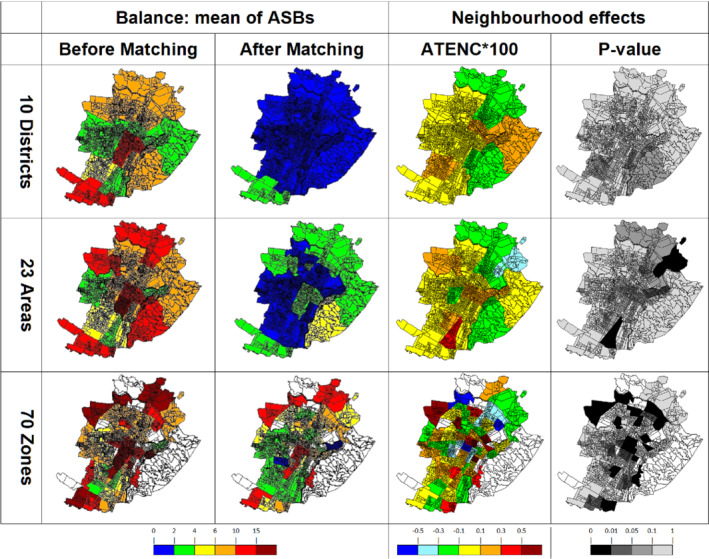
Mean of ASB before and after MARMoT and neighborhood effect estimates (ATENC∗100) for three geographical partitions (10 districts, 23 areas, and 70 zones) and the correspondent *p*‐values. In white excluded zones [Colour figure can be viewed at wileyonlinelibrary.com]

In the first two columns of Figure 1, we plot the mean of the ASB of the variables in each neighborhood before and after the MARMoT procedure in order to visualize areas that are more difficult to balance and those that were more unbalanced in the initial situation. As a general observation, the two areas that are highly unbalanced and difficult to balance are the city center and a neighborhood in the south of Turin, called “Mirafiori Sud”. The composition of “Mirafiori Sud” is quite different from that of the rest of neighborhoods, indeed, in this neighborhood there is a higher percentage of men, individuals born in the South of Italy, subjects with primary or lower education than in the rest of Turin. Moreover, the most common last occupations are home‐makers and laborers.

Considering smaller areas also enabled us to identify neighborhood effects in a greater geographical detail, even though it was more difficult to balance and it proved necessary to discard some of the 70‐zone partition (white area in Figure 1) because they are scarcely populated. Indeed, the eastern part of the city (in white) is hilly and essentially very different and scarcely comparable with the rest of Turin, while the other discarded neighborhoods are mainly occupied by graveyards and factories.

In the population balanced among 10 treatments, 61.92% of the total initial population is included. Subjects excluded are discarded because they were over represented in the initial population and not for lack of overlap. In general, individuals in the balanced population are repeated 1.662 times on average, with a maximum of 32 repetitions. Every neighborhood contains 23 239 subjects after the balancing procedure. Increasing the number of treatments, the percentage of inclusion in the balanced population increases, as the number of times that subjects are repeated (as reported in Table 4).

#### 5.1.4 The size of the total population

In order to assess the importance of the size of the total population in the MARMoT balancing procedure we considered four scenarios by sampling the real population in four different ways: three samples are selected keeping the real distribution of subjects among treatments, but with different sizes (the 75%, 50%, and 25% of the original population); and the fourth sample contains an equal number of subjects in every treatment group (chosen as half of the mean size of neighborhoods in the basis for comparison).

When the population that needs to be balanced decreases, balance reached after MARMoT procedure is worse because it is more difficult to find matches. Subjects are repeated more frequently in the balanced sample in order to overcome this issue and the percentage of individuals included in the final balanced population increases. However, reducing the size of the whole population, it becomes necessary to exclude some profiles (<1%) because they do not belong to the overlapping region of all treatments. However, those few discarded subjects hardly affect final results and estimates of the neighborhood effect.

The scenario where we forced an equal neighborhood size presents a slightly better balance than the scenario with a sample that is half of the whole population, but this corresponds to a slightly better balanced initial situation.

#### 5.1.5 The choice of the frequency reference $f_{r}$ for every row

The choice of the frequency reference $f_{r}$ for every row is a methodological issue that influences the final results of the MARMoT procedure. We have selected the expression (4) to establish $f_{r}$ in order to have a final balanced dataset that presents a size similar to the analyzed population, having as much profiles as possible among those contained in the real population and keeping low the number of subjects repetitions.

Other choices would be less favorable in the delicate trade‐off between the variety of profiles and the repetition of subjects in the final balanced dataset. For instance, when we defined $f_{r}$ as $f_{r}=\text{maximum}(f_{r,1},f_{r,2},\ldots ,f_{r,K})$ (as in the second‐last row of Table 4), the balanced dataset contained more than three times the individuals included in the balanced dataset obtained with the $f_{r}$ definition in formula (4). Moreover, even if the percentage of inclusion is higher than in the basis for comparisons, subjects are repeated 2.758 times on average, with a maximum of 120 times. In this case the balance is not worse than the balance obtained with $f_{r}$ defined as in expression (4). On the other hand, if we define $f_{r}$ as $f_{r}=\text{minimum}(f_{r,1},f_{r,2},\ldots ,f_{r,K})$ (as in the last row of Table 4), several individuals would be discarded because they do not perfectly fit the overlap space between treatments. The balance is perfectly reached, but the final balanced sample would be smaller and far from the real analyzed population, containing less than 10% of it.

### 5.2 The estimation of neighborhood effect

We computed the ATENC for each neighborhood, considering in turn each of the three geographical partitions (full results are collected in Part C of the Supplementary Material). Because fracture is a rare event, we plotted ATENC times 100 in the third column in Figure 1. As a general consideration, if we consider a partition with few big neighborhoods, the estimated effects are more smoothed than in partitions with many small neighborhoods. The fourth column of Figure 1 displays the *p*‐values corresponding to the different estimated ATENCs. The standard errors have been estimated accounting for the repetitions of individuals in the matched dataset. To deal with multiple testing, in the Supplementary Material (Part C) we also indicate whether a False Discovery Rate below certain thresholds (1%, 5%, 10%) is guaranteed or not according to the Benjamini–Hochberg procedure.^38^

In the 23 areas partition, for instance, there is a particularly virtuous neighborhood that is called “Regio–Parco” in the North‐East of Turin (the one in light blue) where the ATENC multiplied by 100 is equal to −0.414 (*p*‐value <0.01). This means that this neighborhood has a protective role in the occurrence of fractures among older individuals reducing, on average, the number of outcomes by one every about 240 residents with respect to residents in the rest of Turin. This neighborhood is also the safest one in the city both in terms of the quality of its streets and because of low crime rate.^16^ On the other hand, the neighborhood that seems to have the most negative impact on the risk of suffering a hospitalized fracture by the older individuals is the “Lingotto”, colored in red, in the South of the city. In fact, on average living in this neighborhood (and not in another one in the rest of the city) increases of one hospitalized fracture every about 265 subjects (ATENC∗100 equal to 0.377 with *p*‐value equal < 0.05).

## 6 CONCLUSIONS

The aim of this paper was to develop and evaluate an original approach, based on poset theory, to balance confounders in a multiple‐treatment framework. The main idea behind our method, that we labeled MARMoT, was to obtain a dataset in which each poset‐based AR value that summarizes the combinations of confounders, was equally represented in all the treatment groups. The MARMoT approach proved very useful in balancing for the confounders. The computation time required is acceptable, even in the case of 70 different treatments. Despite the similarities that our approach shares with template matching, the MARMoT technique is not based on the choice of a template and in the empirical application the final balanced population comprises all the profiles initially included in the population.

Our method enabled us to estimate the neighborhood effect on hospitalized fractures involving older people, considering different geographical partitions (10 districts, 23 smaller areas, and 70 more circumscribed zones) adjusting for the different composition of the neighborhoods. Indeed, once Turin residents over 60 years old residing in different Turin's neighborhoods have a comparable composition with respect to confounders distribution, it is possible to evaluate differences among their distribution of hospitalized fractures. This information will be useful to the Piedmont Region's Epidemiological Service to implement prevention policies for Turin's population and urban interventions focusing on the neighborhoods at greatest risk.

The choice of the geographical scale is a very important issue in neighborhood studies, and several authors have suggested considering different scales, and examining neighborhood effects in more detail, in order to better discern which geographical scale is more relevant to the examined phenomena.^11^ Using our MARMoT method, neighborhood effects can be estimated and compared in different geographical partitions, enabling an assessment of the sensitivity of neighborhood effect estimates to different choices of geographical scale.

Moreover, after the matching step has been implemented with the MARMoT approach, it is possible to implement and perform other analyzes on the balanced datasets, for instance one could use bias correction methods similar to those used after other matching algorithms. In addition, we believe that a promising avenue for future studies is the application of multilevel models, which is a commonly adopted approach in neighborhood studies, on MARMoT‐matched datasets.

Our approach is particularly useful in the presence of a nominal treatment variable with many levels. In fact, our matching approach only exploits the possibility to (partially) order individuals in the space of confounders and, differently from other methods (eg, based on the propensity score) it does not allow per se to exploit the possibility to order the levels of the treatment variable in case of ordinal treatments. Of course, one could exploit the ordered nature of the treatment in the outcome analyzes, that is, in the estimation of causal effects.

As all matching methods, MARMoT may imply discarding units in a treatment group that cannot find a suitable match in other treatment groups (nonoverlap). We suggest the applied research to always inspect the number and characteristics of the discarded units to better understand the subpopulation on which the estimand can be estimated. However, it is worth noticing that in our application we did not discard any unit.

Our results show that when the considered number of treatments is very high (70 in our application), there is still room for improving the MARMoT's performance. As pointed out by our sensitivity analysis, there is a delicate equilibrium between the different choices involved in the implementation of the method. Indeed, reducing the number of variables or levels considered in the balancing procedure allows to take into account a higher number of treatments or a smaller population for the analysis. In future work we plan to examine, with extensive simulation studies, how the choices involved in the implementation of the MARMoT approach affect its performance. This will allow refining an already promising technique.

## CONFLICT OF INTEREST

The authors declare no potential conflict of interests.

## Supporting information


**Data S1** Supplementary Material

## Data Availability

The data that support the findings of this study are available from the Unit “SCaDU Servizio Sovrazonale di Epidemiologia” in Grugliasco (Turin, Italy). Restrictions apply to the availability of these data, which were used under license for this study. Under request, authors may generate a faked dataset as an example.
