# Supplementary material for: Matching on poset‐based average rank for multiple treatments to compare many unbalanced groups
Source: Stat Med. 2021 Sep 16;40(28):6443–58. doi: 10.1002/sim.9192 (PMC9292765; doi:10.1002/sim.9192)
Supplement: Supplementary file 1 — Data S1 Supplementary Material [file SIM-40-6443-s001.pdf]

Received: Added at production | Revised: Added at production | Accepted: Added at production
DOI: xxx/xxxx

ARTICLE TYPE

# Supplementary on line material for the paper "Matching on poset-based Average Rank for Multiple Treatments (MARMoT) to compare many unbalanced groups"

Margherita Silan*[1] | Giovanna Boccuzzo[1] | Bruno Arpino[2]

[1]Department of Statistical Sciences, University of Padua, Padua, Italy
[2]Department of Statistics, Computer Science, Applications, University of Florence, Florence, Italy

**Correspondence**
*Margherita Silan Email: silan@stat.unipd.it

**Summary**

In this document we provide additional material for the paper "Matching on poset-based Average Rank for Multiple Treatments (MARMoT) to compare many unbalanced groups". Section A provides some details about Partially Ordered Set theory, with an explicative example and some specifics about the estimation of the average rank. Section B provides the R code used in the simulations. Section C collects all ATENC estimates and correspondent p-values for three geographical partitions (10 districts, 23 areas and 70 zones).

## A - POSET THEORY AT A GLANCE

### A.1 - Introduction to poset theory

A partially ordered set (poset) is, in mathematics, a set of elements where a binary relation that indicates an order can be traced; the word “partially” refers to the fact that not every pair of elements is comparable. Poset theory is a theoretical field between graph theory and discrete mathematics that quickly developed after the 1970s thanks to technological advances that made greater computational efforts manageable[1].

When dealing with a population, the people comprising it can be ranked and ordered using a single variable: level of education, for instance, enables two different individuals to be arranged in an order. From the mathematical standpoint, an order is a binary relation between the elements in a set that respects specific properties. Let $P$ be a set, an order on $P$ is a relation ($\leq$) between two elements in the set $P$ such that, for all $x, y, z \in P$, the following properties hold:

- Reflexivity: $x \leq x$
- Antisymmetry: $x \leq y$ and $x \geq y$ implies $x = y$
- Transitivity: $x \leq y$ and $y \leq z$ implies $x \leq z$.

A set equipped with such a relation is said to be ordered. If the comparison is drawn using several variables, it may be that some elements are neither equal nor ordered, in which case they are defined as incomparable[2]. The word “partially” is added to “ordered set” when some of its elements are incomparable, so the order relation has to be changed to a partial order relation, which takes the incomparability (indicated with $||$) of the elements into account:

Incomparability: $x||y \leftrightarrow x \nleq y \quad and \quad y \nleq x, \quad x, y \in P$ .

Comparing the individuals in a population gives rise to a list of comparabilities and incomparabilities, which can be represented in a graphic form called a Hasse diagram. This diagram represents the elements in a poset: each node is an element, two

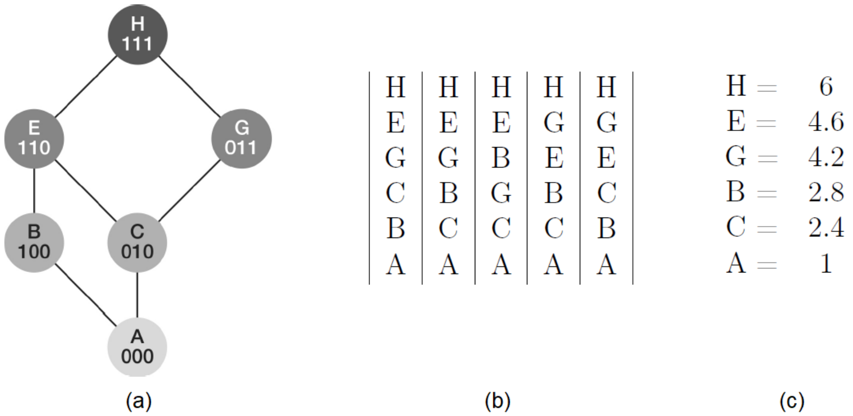


**FIGURE 1** A poset and its linear extensions: part (a) represents the Hasse diagram for the individuals in Table 1; part (b) lists all the linear extensions for these individuals; and part (c) their exact average rank.

or more equal elements still form one node, and every line segment is an order relation between comparable objects. Let us suppose that we have a population comprising six individuals characterized by three dichotomous variables, as represented in Table 1: age (which takes a value of 0 for individuals who are between 60 and 70 years old, and 1 if they are older); education (which takes a value of 0 if they have a higher education, and 1 otherwise); and homeowner (which takes a value of 0 if they own the house in which they live, and 1 otherwise). The set of observed characteristics of each individual is called "profile". These variables are ordered according to the risk of experiencing the outcome.

**TABLE 1** Toy example for a group of observations.

| Subject | Age | Education | Homeowner |
|---|---|---|---|
| A | 0 | 0 | 0 |
| B | 1 | 0 | 0 |
| C | 0 | 1 | 0 |
| E | 1 | 1 | 0 |
| G | 0 | 1 | 1 |
| H | 1 | 1 | 1 |

In this example, for the sake of simplicity, we included only dichotomous variables, but categorical and discrete variables may be also considered in a poset. However, in order to contain the complexity of the poset, it is recommended to reduce each discrete variable in few meaningful classes.

A Hasse diagram can be used to visualize the order relations between the elements in a poset, and it is based entirely on the order of the elements, disregarding any quantitative information.

In Figure 1(a), the six individuals are represented by their profile in the Hasse diagram, where each node stands for a profile. When two individuals are comparable, they are connected by line segments in the diagram, like A and B (where A and B have same values for education and homeowner, and B has a higher value for age than A) or B and E (where B and E have same values for age and homeowner, and E has a higher value for education than B), whereas there is no ascending or descending path between incomparable elements, like B and C (where B and C have same value for homeowner, B has a higher value for age than C, but a lower value for education).

The list of all the ranks that each individual may occupy is shown in part (b) of Figure 1, where all the linear extensions of the poset are listed. Linear extensions are all the possible rankings of elements in the poset that respect its comparabilities (the connections in the Hasse diagram) and incomparabilities [1,2]. The average rank (AR) of a node represents the mean of all the ranks that the element occupies in all possible linear extensions, starting from the known order relations, as listed in Figure 1 part (c).

The AR is a single value for each element in the set that describes the relative position of a given element with respect to the rest of the population. It can be normalized in the interval [0;1].

## A.2 - Approximating the average rank

If the number of individuals and variables increases, the linear extensions become too many to be examined thoroughly, and it becomes computationally almost impossible to find the exact AR as in the example in Table 1. That said, satisfactory approximations of the number of linear extensions of a poset can be found in works by Dyer[3], and De Loof[4].

Researchers have used two main approaches to obtain a computationally efficient calculation of the AR, by sampling linear extensions[5,6], or defining an approximation formula. Different approximation formulas have been proposed in the literature, such as the Local Partial Order Model[7], or the one based on Mutual Probabilities[4]. The present work is based on De Loof's approach (2009)[8] because it provides better results than other methods in terms of accuracy with a large sample size[8].

Two concepts help us to understand this approximation, for a sample $P$ with $|P|$ elements:

**The rank probability** $P(rank(x) = i)$ is the fraction of linear extensions in which an element's rank equals $i$, where $i$ assumes the value of all possible ranks in the sample of size $|P|$, so $i = 1, \dots, |P|$.

**The mutual rank probability** $P(x > y)$ of two elements $x, y \in P$ is the fraction of linear extensions in which the element $x$ is ranked higher than element $y$.

Now we can establish a relation between the last-mentioned two concepts and the real AR of elements $x$, $\bar{h}(x)$, starting from a sample $P$ with $|P|$ elements, including $x$ and $y$:

$$\bar{h}(x) = \sum_{i=1}^{|P|} i \cdot P(rank(x) = i) = 1 + \sum_{y=1}^{|P|} P(x > y). \tag{1}$$

In other words, the first part of formula 1 describes the real AR value, $\bar{h}(x)$, as the expected value, multiplying each possible rank value $i$ by the fraction of linear extensions in which the element's rank equals $i$. The second part of formula 1 expresses the real AR value as the sum of all the mutual rank probabilities that involve the element $x$. Starting from this formula, we need to find an approximation for the mutual rank probability. To do so, we have to define three subsets of the poset P, given a generic element $x \in P$:

**Downset:** $O(x) = \{y \in P : y \leq x\}$;

**Upset:** $F(x) = \{y \in P : y \geq x\}$;

**Incomparables:** $U(x) = \{y \in P : y || x\}$

If $y \in O(x)$, then $P(rank(x) > rank(y))$ equals 1, and if $y \in F(x)$, then $P(rank(x) > rank(y))$ equals 0, so the mutual rank probabilities only need to be approximated with respect to the reciprocal ranks of the incomparable elements. The following approximation was proposed by Brüggemann[9]

$$P^*(x > y) = \frac{[o(x) + 1][f(y) + 1]}{[o(x) + 1][f(y) + 1] + [o(y) + 1][f(x) + 1]}, \tag{2}$$

where $o(x) = |O(x) \setminus \{x\}|$ and $f(x) = |F(x) \setminus \{x\}|$ are respectively the number of elements in the downset and the upset of $x$ without $\{x\}$. Two more quantities are needed to approximate the AR according to the De Loof[4] formula, $\tilde{o}(x)$ and $\tilde{f}(x)$:

$$\tilde{o}(x) = o(x) + \sum_{y \in U(x)} P^*(x > y) \quad and \tag{3}$$

$$\tilde{f}(x) = f(x) + \sum_{y \in U(x)} P^*(x < y), \tag{4}$$

and the AR approximation proposed by De Loof[4] is

$$AR(x) = o(x) + 1 + \sum_{y \in U(x)} \frac{[\tilde{o}(x) + 1][\tilde{f}(y) + 1]}{[\tilde{o}(x) + 1][\tilde{f}(y) + 1] + [\tilde{o}(y) + 1][\tilde{f}(x) + 1]}. \tag{5}$$

That is to say that using formula 5, the AR of $x$ is given by the number of elements in its downset and the sum of probabilities of being a part of $x$'s downset for all incomparable elements with respect to $x$, using the approximation of the *mutual rank*

*probabilities*. Following the toy example in Table 1, the steps needed to approximate the AR with the De Loof[4] approach are solved in Table 2, including the estimation of the AR.

**TABLE 2** A numerical example of the approximation of the average rank according to De Loof[4] approach.

| $x$ | $o(x)$ | $f(x)$ | U(x) | $Pr^*$ $(x > y)$ | | | | | | $\tilde{o}(x)$ | $\tilde{f}(x)$ | AR(x) |
|---|---|---|---|---|---|---|---|---|---|---|---|---|
| | | | | $y = A$ | $y = B$ | $y = C$ | $y = E$ | $y = G$ | $y = H$ | | | |
| A | 0 | 5 | 0 | . | 0.20 | 0.25 | 0.08 | 0.10 | 0.03 | 0.00 | 5.00 | 1.00 |
| B | 1 | 2 | C, G | 0.80 | . | 0.57 | 0.25 | 0.31 | 0.10 | 1.88 | 3.12 | 2.90 |
| C | 1 | 3 | B | 0.75 | 0.43 | . | 0.20 | 0.25 | 0.08 | 1.43 | 3.57 | 2.43 |
| E | 3 | 1 | G | 0.92 | 0.75 | 0.80 | . | 0.57 | 0.25 | 3.57 | 1.43 | 4.57 |
| G | 2 | 1 | B, E | 0.90 | 0.69 | 0.75 | 0.43 | . | 0.20 | 3.12 | 1.88 | 4.10 |
| H | 5 | 0 | 0 | 0.97 | 0.90 | 0.92 | 0.75 | 0.80 | . | 5.00 | 0.00 | 6.00 |

In the present work, the approximated AR was computed using the R software, with an R function, called `deloof`, proposed by Caperna[10,11] that can cope with large datasets[12,13].

## B - R CODE FOR THE SIMULATION STUDY

```
Required( parsec, dplyr, multiwayvcov, lmtest)
### Computation of the average rank using Caperna (2009) approximation function implemented in R,
where set is a matrix that contains all observable confounders (columns) for all the considered
subjects (rows)
ar<-deloof(set)
###Normalization of the average rank value
ar_norm<-(ar-min(ar))/(max(ar)-min(ar))
data$ar<-ar_norm

### Set the number of iterations
num<-1000

### Need the data matrix that contains all individual characteristics, personal ids and the
computed average rank; and the prob.sim matrix that contains the computed probabilities
for each subject to be assigned to each one of the 23 treatments (according to one scenario)

### Create matrices to save measure of balance and computational time
ASB_pre<-matrix(data=NA, nrow=num, ncol=8)
ASB_post<-matrix(data=NA, nrow=num, ncol=8)
time_bal<-c()

### Starting with the simulation
for (k in 1:num){
### Simulation of the treatment
ass.treat = t(apply(prob.sim, 1, rmultinom, n = 1, size = 1))
s1 = cbind.data.frame(data, treat_sim = apply(ass.treat, 1, function(x) which(x==1)))

### Saving balance measure relative to the population before balance
pre<-table(s1$sex, s1$treat_sim)
```

```
pre<-rbind(pre,table(s1$age, s1$treat_sim))
pre<-rbind(pre,table(s1$edu, s1$treat_sim))
pre<-rbind(pre,table(s1$fami, s1$treat_sim))
pre<-rbind(pre,table(s1$occ, s1$treat_sim))
pre<-rbind(pre,table(s1$birth_reg, s1$treat_sim))
pre<-rbind(pre,table(s1$homeowner, s1$treat_sim))
perc_treatm<-matrix(data=NA, nrow = 24, ncol = 23)
for (i in 1:23){
perc_treatm[,i]<-pre[-c(1,25),i]/tab_treats[k,i]}
var_treatm<-perc_treatm*(1-perc_treatm)
perc_tot<-apply(pre[-c(1,25),],1,sum)/(sum(tab_treats[k,]))
var_tot<-perc_tot*(1-perc_tot)
t_asb_pre<-matrix(data=NA,nrow=24, ncol = 23)
for (i in 1:24){
t_asb_pre[i,]<-(abs(perc_treatm[i,]-perc_tot[i]))/(sqrt((var_treatm[i,]+var_tot[i])/2))*100}
ASB_pre[k,]<-c(quantile(t_asb_pre, probs = c(0, 0.25, 0.5, 0.75, 1)), mean(t_asb_pre),
length(which(t_asb_pre>5)), length(which(t_asb_pre>10)))

### Preparation of the frequency table and setting of other parameters needed in the balancing
procedure
freq<-table(s1$ar, s1$t)
ps<-sort(unique(s1$ar))
freq<-cbind(ps, freq)
### Set the caliper to define the tolerance interval
caliper<-sd(s1$ar)/4
n<-as.numeric(colnames(freq)[-1]) # Identification codes of treatment groups considered
nT<-length(n)  # Number of treatments in the matching procedure
### Set the frequency reference for each row fr
ref<-ifelse(ceiling(apply(freq[,-1], 1, median))==0,1,ceiling(apply(freq[,-1], 1, median)))

### Create empty vectors to store individual identification codes that will be included in
the balanced population
new<-c()
new0<-c()
sub0<-c()
rem<-c()

### Start the balancing procedure
start<-Sys.time()
### Consider every column separately
for (i in 1:nT){
same<- freq[,i+1]==ref
zero<-freq[,i+1]==0
different<-freq[,i+1]>0 & freq[,i+1]!=ref

ok<-rep(0, dim(s1)[1])
ok<-ifelse(s1$ar %in% freq[same==TRUE,1] & s1$t==n[i], TRUE, FALSE)
new<-c(new, which(ok))

for (j in which(different)){
cond_tosample<-which(ifelse(s1$ar==freq[j,1] & s1$t==n[i], TRUE, FALSE))
```

```
if (length(cond_tosample)==1){
ok<-rep(cond_tosample, ref[j])   }
if (length(cond_tosample)>1){
ok<-sample(cond_tosample, ref[j], replace = TRUE) }
new<-c(new, ok)}

for (j in which(zero)){
diff<-abs(freq[!zero,1]-freq[j,1])
value<-ifelse(sort(diff)[1]<=caliper,as.numeric(names(sort(diff))[1]),-1)
if (value==-1){rem<-c(rem,freq[j,1])}
if (value!=-1){
cond_tosample<-which(ifelse(s1$ar==value & s1$t==n[i], TRUE, FALSE))
if (length(cond_tosample)==1){
ok<-rep(cond_tosample, ref[j])
}
if (length(cond_tosample)>1){
ok<-sample(cond_tosample, ref[j], replace = TRUE)
}
new0<-c(new0, ok)
sub0<-c(sub0, rep(freq[j,1], ref[j]))
}}}
end<-Sys.time()

###build the balanced population
balanced_pop<-s1[new,]
balanced_pop0<-s1[new0,]
balanced_pop0$ar2<-sub0
after_all<-rbind(balanced_pop, balanced_pop0)
after<-after_all[!(after_all$ar2 %in% rem), ]

########save measure of balance on the balanced population
post<-table(after$sex, after$t)
post<-rbind(post,table(after$age, after$t))
post<-rbind(post,table(after$edu, after$t))
post<-rbind(post,table(after$fami, after$t))
post<-rbind(post,table(after$occ, after$t))
post<-rbind(post,table(after$birth_reg, after$t))
post<-rbind(post,table(after$homeowner, after$t))
treatm<-as.numeric(table(after$t))[1]
perc_treatm<-post[-c(1,25),]/treatm
var_treatm<-perc_treatm*(1-perc_treatm)
perc_tot<-apply(post[-c(1,25),],1,sum)/(treatm*23)
var_tot<-perc_tot*(1-perc_tot)
t_asb<-matrix(data=NA,nrow=24, ncol = 23)
for (i in 1:24){
t_asb[i,]<-(abs(perc_treatm[i,]-perc_tot[i]))/(sqrt((var_treatm[i,]+var_tot[i])/2))*100  }
time_bal[k] <-end-start
ASB_post[k,]<-round(c(quantile(t_asb, probs = c(0, 0.25, 0.5, 0.75, 1)), mean(t_asb),
length(which(t_asb>5)), length(which(t_asb>10))), digits=3)
```

## C - ATENC ESTIMATES AND CORRESPONDENT P-VALUES FOR THREE GEOGRAPHICAL PARTITIONS (10 DISTRICTS, 23 AREAS AND 70 ZONES)

In the table below, we report the ATENC estimates and correspondent p-values for three geographical partitions (10 districts 3, 23 areas 4 and 70 zones 5). Moreover, asterisks indicate whether a False Discovery Rate below certain thresholds (1%, 5%, 10%) is guaranteed or not according to the Benjamini-Hochberg procedure [14].

**TABLE 3** ATENC estimates and correspondent p−values and False Discovery Rate (FDR). The last column indicates whether a False Discovery Rate below certain thresholds (1%, 5%, 10%) is guaranteed or not according to the Benjamini-Hochberg procedure.

| Neighbourhood | ATENC*100 | P-value | FDR |
|---|---|---|---|
| 1 Centro, Crocetta | 0,044 | 0,495 | |
| 2 Santa Rita, Mirafiori nord | 0,159 | 0,014 | * |
| 3 San Paolo, Cenisia, Cit Turin, Pozzo Strada | -0,061 | 0,342 | |
| 4 San Donato, Parella, Campidoglio | 0,015 | 0,812 | |
| 5 Borgo Vittoria, Madonna di Campagna, Lanzo, Lucento, Vallette | 0,011 | 0,870 | |
| 6 Regio Parco, Barca, Bertolla, Barriera di Milano, Rebaudengo, Falchera, Villaretto | -0,104 | 0,106 | |
| 7 Aurora, Vanchiglia, Sassi, Madonna del Pilone | 0,125 | 0,052 | |
| 8 Borgo Po, San Salvario, Cavoretto | -0,152 | 0,018 | * |
| 9 Nizza Millefonti, Lingotto, Filadelfia | -0,028 | 0,667 | |
| 10 Mirafiori sud | -0,009 | 0,894 | |

**TABLE 4** ATENC estimates and correspondent p−values and False Discovery Rate (FDR). The last column indicates whether a False Discovery Rate below certain thresholds (1%, 5%, 10%) is guaranteed or not according to the Benjamini-Hochberg procedure.

| Neighbourhood | ATENC*100 | P-value | FDR |
|---|---|---|---|
| 1 Centro | 0,196 | 0,030 | * |
| 2 S. Salvario | 0,062 | 0,491 | |
| 3 Crocetta | -0,081 | 0,370 | |
| 4 S.Paolo | -0,043 | 0,636 | |
| 5 Cenisia | -0,233 | 0,010 | * |
| 6 S.Donato | 0,062 | 0,491 | |
| 7 Aurora | -0,043 | 0,636 | |
| 8 Vanchiglia | 0,253 | 0,005 | ** |
| 9 Nizza Millefonti | -0,005 | 0,960 | |
| 10 Lingotto | 0,377 | 0,000 | *** |
| 11 S.Rita | 0,205 | 0,023 | * |
| 12 Mirafiori Nord | 0,062 | 0,491 | |
| 13 Pozzo Strada | -0,081 | 0,370 | |
| 14 Parella | -0,024 | 0,793 | |
| 15 Lucento Vallette | 0,186 | 0,039 | |
| 16 M.Campagna Lanzo | -0,024 | 0,793 | |
| 17 Borgo Vittoria | -0,033 | 0,713 | |
| 18 Barriera di Milano | -0,205 | 0,023 | * |
| 19 Rebaudengo Falchera | -0,214 | 0,018 | * |
| 20 Regio Parco Barca | -0,414 | 0,000 | *** |
| 21 Madonna del Pilone | 0,015 | 0,872 | |
| 22 Cavoretto Borgo Po | -0,109 | 0,225 | |
| 23 Mirafiori Sud | 0,091 | 0,314 | |

**TABLE 5** ATENC estimates and correspondent p−values and False Discovery Rate (FDR). The last column indicates whether a False Discovery Rate below certain thresholds (1%, 5%, 10%) is guaranteed or not according to the Benjamini-Hochberg procedure.

| Neighbourhood | ATENC*100 | P-value | FDR |
|---|---|---|---|
| 01-Municipio | 0,579 | 0,000 | *** |
| 03-Palazzo Carignano | -0,204 | 0,084 | |
| 04-Piazza San Carlo - Piazza Carlo Felice | -0,493 | 0,000 | *** |
| 05-Piazza Statuto | 0,409 | 0,001 | *** |
| 06-Piazza Vittorio Veneto | -0,051 | 0,668 | |
| 07-Borgo Nuovo | 0,120 | 0,310 | |
| 08-Comandi militari | -0,510 | 0,000 | *** |
| 09-San Salvario | 0,664 | 0,000 | *** |
| 10-Porta Nuova - San Secondo | -0,306 | 0,009 | ** |
| 11-Vanchiglia | 0,460 | 0,000 | *** |
| 12-Borgo Dora | 0,103 | 0,384 | |
| 13-Parco Michelotti - Borgo Po | -0,136 | 0,249 | |
| 14-Motovelodromo | 0,017 | 0,882 | |
| 16-San Donato | 0,222 | 0,060 | |
| 17-Porta Susa - Nuovo Tribunale | -0,306 | 0,009 | ** |
| 18-Politecnico | 0,290 | 0,014 | ** |
| 19-Piazza Nizza | 0,000 | 0,997 | |
| 20-Corso Dante - Ponte Isabella | 0,154 | 0,192 | |
| 22-Vanchiglietta | 0,034 | 0,769 | |
| 23-Rossini | 0,579 | 0,000 | *** |
| 24-Aurora | -0,136 | 0,249 | |
| 25-Teksid - Ospedale Amedeo di Savoia | -0,238 | 0,043 | |
| 26-Crocetta | 0,034 | 0,769 | |
| 27-Ospedale Mauriziano | 0,205 | 0,082 | |
| 28-Corso Lepanto | -0,255 | 0,030 | * |
| 29-Campidoglio | 0,017 | 0,882 | |
| 30-La Tesoriera | 0,051 | 0,662 | |
| 31-Boringhieri | -0,102 | 0,388 | |
| 32-Cenisia | -0,255 | 0,030 | * |
| 33-San Paolo | -0,306 | 0,009 | ** |
| 34-Monginevro | -0,187 | 0,113 | |
| 35-Polo Nord | -0,068 | 0,566 | |
| 37-Maddalene | 0,000 | 0,997 | |
| 38-Monterosa | -0,085 | 0,472 | |
| 39-Monte Bianco | -0,408 | 0,001 | *** |
| 40-Regio Parco | -0,595 | 0,000 | *** |
| 41-Barriera di Milano | -0,323 | 0,006 | ** |
| 42-Borgata Vittoria | 0,000 | 0,997 | |
| 43-La Fossata | 0,477 | 0,000 | *** |
| 44-Officine Savigliano | -0,187 | 0,113 | |
| 45-Madonna di Campagna | -0,204 | 0,084 | |
| 46-Barriera di Lanzo | -0,204 | 0,084 | |
| 47-Ceronda-Martinetto | 0,681 | 0,000 | *** |
| 48-Lucento | -0,034 | 0,776 | |
| 50-Parella - Lionetto | 0,137 | 0,246 | |
| 51-Pozzo Strada | -0,153 | 0,195 | |
| 52-Parco Ruffini - Borgata Lesna | 0,171 | 0,147 | |
| 53-Santa Rita | 0,256 | 0,030 | * |
| 54-Stadio Comunale - Piazza d'Armi | 0,426 | 0,000 | *** |
| 55-Istituto di Riposo per la Vecchiaia | 0,000 | 0,997 | |
| 56-Mercati generali | 0,239 | 0,043 | |
| 57-Molinette - Millefonti | -0,289 | 0,014 | ** |
| 58-Lingotto - Barriera di Nizza | -0,102 | 0,388 | |
| 59-Corso Siracusa | 0,120 | 0,310 | |
| 60-Fiat Mirafiori | -0,119 | 0,314 | |
| 61-Corso Traiano | 0,034 | 0,769 | |
| 62-Gerbido | -0,034 | 0,776 | |
| 63-Venchi Unica | 0,154 | 0,192 | |
| 64-Aeronautica | -0,153 | 0,195 | |
| 65-Le Vallette | 0,647 | 0,000 | *** |
| 66-Strada di Lanzo | -0,697 | 0,000 | *** |
| 68-Barriera di Stura | -0,408 | 0,001 | *** |
| 70-Pilonetto | 0,358 | 0,002 | *** |
| 72-Sassi | -0,187 | 0,113 | |
| 77-Falchera | 0,137 | 0,246 | |
| 78-Villaggio Snia - Abbadia di Stura | -0,221 | 0,061 | |
| 79-Bertolla | -0,102 | 0,388 | |
| 89-Giardino Colonnetti | 0,477 | 0,000 | *** |
| 90-Borgata Mirafiori | -0,289 | 0,014 | ** |
| 91-Drosso | 0,086 | 0,467 | |

# References


1. Brüggemann R, Patil GP. *Ranking and prioritization for multi-indicator systems: Introduction to partial order applications*. Springer Science & Business Media . 2011.

2. Davey BA, Priestley HA. *Introduction to lattices and order*. Cambridge university press . 2002.

3. Dyer M, A. F, R. K. A random polynomial-time algorithm for approximation the volume of convex bodies. *Journal of the ACM* 1991; 38(1): 1-17.

4. De Loof K. *Efficient computation of rank probabilities in posets*. PhD thesis. Ghent University, 2009. (Available from https://biblio.ugent.be/publication/874495).

5. Fattore M. Partially ordered sets and the measurement of multidimensional ordinal deprivation. *Social Indicators Research* 2016; 128(2): 835-858.

6. Lerche D, Sorensen P. Evaluation of the ranking probabilities for partial orders based on random linear extensions. *Chemosphere* 2003; 53: 981–992.

7. Brüggemann R, Carlsen L. An Improved Estimation of Averaged Ranks of Partial Orders. *MATCH Communications in Mathematical and in Computer Chemistry* 2011; 65: 383-414.

8. De Loof K, De Baets B, De Meyer H. Approximation of average ranks in posets. *MATCH Communications in Mathematical and in Computer Chemistry* 2011; 66: 219-229.

9. Brüggemann R, Lerche D, Sorensen PB. First attempts to relate structures of Hasse diagrams with mutual probabilities. *Order Theory in Environmental Sciences* 2004.

10. Caperna G. Approximation of AverageRank by means of a formula. 2019. Doi: https://doi.org/10.5281/zenodo.2565699

11. Caperna G. *Partial Order Theory for Synthetic Indicators*. PhD thesis. University of Padova, 2016. (Available from http://paduaresearch.cab.unipd.it/9588/).

12. Boccuzzo G, Caperna G. Evaluation of Life Satisfaction in Italy: Proposal of a Synthetic Measure Based on Poset Theory. In: Springer. 2017 (pp. 291-321).

13. Caperna G, Boccuzzo G. Use of Poset Theory with Big Datasets: A New Proposal Applied to the Analysis of Life Satisfaction in Italy. *Social Indicators Research* 2018; 136(3): 1071-1088.

14. Benjamini Y, Hochberg Y. Controlling the false discovery rate: a practical and powerful approach to multiple testing. *Journal of the Royal statistical society: series B (Methodological)* 1995; 57(1): 289-300.


□
